# Lightweight Evolving U-Net for Next-Generation Biomedical Imaging

**DOI:** 10.3390/diagnostics15091120

**Published:** 2025-04-28

**Authors:** Furkat Safarov, Ugiloy Khojamuratova, Misirov Komoliddin, Ziyat Kurbanov, Abdibayeva Tamara, Ishonkulov Nizamjon, Shakhnoza Muksimova, Young Im Cho

**Affiliations:** 1 Department of Computer Engineering, Gachon University, Sujeong-gu, Seongnam-si 461701, Republic of Korea; safarov@gachon.ac.kr (F.S.); shakhnoza02@gachon.ac.kr (S.M.); 2 Department of Computer Science, CUNY Queens College, 65-30 Kissena Blvd Flushing, New York, NY 11374, USA; ugiloy.khojamuratova48@qmail.cuny.edu; 3 Department of Financial Accounting and Reporting, Tashkent State University of Economics, Tashkent 100066, Uzbekistan; misirovkomoliddin0218@tsue.uz (M.K.); ziyatkurbanov@tsue.uz (Z.K.); abdibayevatamara@tsue.uz (A.T.); 4 Department of Banking and Accounting, Kimyo International University in Tashkent, Tashkent 100121, Uzbekistan; n.ishonkulov@kiut.uz

**Keywords:** medical image segmentation, biomedical image analysis, computational efficiency, nuclei segmentation

## Abstract

**Background/Objectives**: Accurate and efficient segmentation of cell nuclei in biomedical images is critical for a wide range of clinical and research applications, including cancer diagnostics, histopathological analysis, and therapeutic monitoring. Although U-Net and its variants have achieved notable success in medical image segmentation, challenges persist in balancing segmentation accuracy with computational efficiency, especially when dealing with large-scale datasets and resource-limited clinical settings. This study aims to develop a lightweight and scalable U-Net-based architecture that enhances segmentation performance while substantially reducing computational overhead. **Methods**: We propose a novel evolving U-Net architecture that integrates multi-scale feature extraction, depthwise separable convolutions, residual connections, and attention mechanisms to improve segmentation robustness across diverse imaging conditions. Additionally, we incorporate channel reduction and expansion strategies inspired by ShuffleNet to minimize model parameters without sacrificing precision. The model performance was extensively validated using the 2018 Data Science Bowl dataset. **Results**: Experimental evaluation demonstrates that the proposed model achieves a Dice Similarity Coefficient (DSC) of 0.95 and an accuracy of 0.94, surpassing state-of-the-art benchmarks. The model effectively delineates complex and overlapping nuclei structures with high fidelity, while maintaining computational efficiency suitable for real-time applications. **Conclusions**: The proposed lightweight U-Net variant offers a scalable and adaptable solution for biomedical image segmentation tasks. Its strong performance in both accuracy and efficiency highlights its potential for deployment in clinical diagnostics and large-scale biological research, paving the way for real-time and resource-conscious imaging solutions.

## 1. Introduction

The accurate and efficient segmentation of cell nuclei in biomedical images represents a cornerstone in computational pathology and quantitative histomorphometry [1]. This task underpins a multitude of critical downstream applications, including cancer diagnosis, tumor grading, cell classification, and drug response prediction [2]. The precision with which nuclei are segmented directly impacts the reliability of diagnostic interpretations and prognostic outcomes [3], making this an indispensable function in digital pathology workflows [4].

Traditional segmentation techniques—such as thresholding algorithms [5], morphological operations [6], and marker-controlled watershed transformations [7]—have demonstrated limited robustness in complex imaging environments characterized by variable cell morphology, staining inconsistencies, overlapping structures, and low signal-to-noise ratios. Machine learning-based classifiers such as Support Vector Machines and Random Forests [8,9,10,11] improved upon classical methods by leveraging hand-crafted features, yet remained constrained by poor generalizability and the necessity for domain-specific feature engineering [12,13]. The paradigm shifted with the introduction of Convolutional Neural Networks (CNNs), which enabled end-to-end learning of hierarchical features directly from raw pixel data. Among these, the U-Net architecture [14] has emerged as the gold standard in biomedical image segmentation due to its elegant encoder–decoder structure and use of skip connections to preserve spatial resolution during downsampling and upsampling operations. U-Net and its variants have been widely adopted for nuclei segmentation tasks, achieving superior results compared to conventional approaches [15,16,17,18]. Despite its success, the standard U-Net model suffers from significant computational overheads due to its deep architecture and large number of parameters. These limitations are particularly problematic in clinical environments where real-time inference, scalability, and deployment on hardware-constrained systems are essential. To address these challenges, several extensions to U-Net have been proposed, including architectures enhanced with residual learning [19], attention mechanisms [20], multi-resolution feature integration [21,22], and channel reduction strategies inspired by lightweight CNNs such as ShuffleNet [23]. However, many of these extensions either increase the architectural complexity or exhibit trade-offs between segmentation accuracy and computational efficiency. A practical solution requires a model that not only delivers state-of-the-art performance but also reduces memory usage, training time, and inference latency without sacrificing precision. In this study, we present a novel architecture—Lightweight Evolving U-Net—designed to address these pressing challenges. Our model incorporates:

- Multi-scale feature extraction to improve the capture of nuclei with varying size and morphology.
- Depthwise separable convolutions to reduce parameter count and computational burden.
- Residual connections to facilitate deeper learning and stable convergence.
- Channel reduction and expansion blocks inspired by ShuffleNet for optimized memory usage.
- Spatial attention mechanisms to refine feature relevance and suppress background noise in cluttered histopathological scenes.

We evaluate our model on the 2018 Data Science Bowl dataset [24], which includes a wide range of cell types, imaging conditions, and magnification levels. The results demonstrate that our model achieves a Dice Similarity Coefficient (DSC) of 0.95 and an accuracy of 0.94, outperforming both the baseline and state-of-the-art segmentation architectures in terms of precision and efficiency. Our proposed method offers a scalable, lightweight, and high-performance solution for nuclei segmentation, advancing the field of deep learning in biomedical imaging and supporting the broader adoption of AI-powered tools in clinical and research environments.

## 2. Related Works

The segmentation of cell nuclei has long been a critical objective in biomedical image analysis due to its direct relevance to disease classification, tissue morphology quantification, and cellular phenotyping [25]. Early approaches primarily relied on intensity-based thresholding techniques such as Otsu’s method [5] and watershed algorithms [7], which, while computationally inexpensive [26], suffered from poor performance in the presence of uneven illumination, overlapping nuclei, and variable staining intensities [27]. The advent of machine learning introduced a new paradigm [28], where traditional classifiers such as Support Vector Machines (SVMs) and Random Forests were trained on hand-crafted features to distinguish nuclei from the background [8,9,10,11]. Despite their improved adaptability, these methods were inherently constrained by the manual feature engineering process and often lacked the flexibility required to generalize across diverse imaging conditions [12,13].

A transformative shift occurred with the introduction of CNNs, most notably the U-Net architecture [14], which has since become the de facto standard in biomedical image segmentation. U-Net’s design—a symmetric encoder–decoder structure with skip connections—enables the model to capture high-resolution spatial features while maintaining contextual awareness. This architecture laid the foundation for numerous variants aiming to address specific challenges in segmentation accuracy and computational demand. Among these, V-Net [29] extended U-Net’s capabilities to volumetric data, making it suitable for 3D medical imaging tasks. ResUNet [19,30] introduced residual connections to improve the gradient flow and convergence speed, while Attention U-Net [20] integrated spatial attention gates to emphasize biologically relevant structures, proving effective in cluttered or low-contrast images. Efforts to reduce computational complexity led to the development of lightweight architectures such as GA-UNet [20], which employs Ghost modules and attention bottlenecks, and SACU-Net [31], which combines attention and context modules in a shape-aware U-Net structure [32]. These models achieve impressive segmentation results while significantly reducing the number of parameters, a crucial factor for deployment in real-time or embedded systems. Further, nnU-Net [33] introduced an automated configuration strategy that adapts the network’s parameters and architecture based on the dataset properties, demonstrating robust cross-dataset generalization. Simultaneously, DeepLab V3+ [34], with its use of atrous spatial pyramid pooling (ASPP), provides improved multi-scale feature extraction but remains relatively heavy in terms of computational cost. Recent literature also includes NuSEA [29], which utilizes elliptical annotations for better geometric fitting of nuclei, and R2U-Net [19], which merges recurrent and residual layers for refined feature aggregation. Generative Adversarial Networks (GANs) have been employed in segmentation tasks to improve robustness through adversarial learning [35], while domain adaptation techniques [36] are being explored to reduce the need for annotated data in new imaging modalities [37]. Despite these advances, achieving optimal performance across diverse datasets with varying cell types and imaging conditions remains challenging. Many state-of-the-art (SOTA) models either prioritize accuracy at the cost of efficiency or simplify architectures in ways that degrade performance. Our proposed Lightweight Evolving U-Net addresses this trade-off by integrating depthwise separable convolutions, spatial attention, channel compression and expansion blocks, and residual learning—all within a scalable encoder–decoder framework. By synthesizing the most effective innovations across SOTA models [14,19,20,29,31,33,34,38], our architecture delivers high segmentation fidelity with a reduced computational cost, making it particularly suitable for real-time clinical and research applications.

## 3. Materials and Methods

In this work, we introduce one of the hot topics in the DNA world, the segmentation of the nuclei, as the accurate identification of cell nuclei serves as the foundational step in most analytical workflows, given that the majority of the human body’s approximately 30 trillion cells house a nucleus containing DNA, the genetic blueprint that governs cellular function. Delineating nuclei enables precise cell segmentation within a sample, facilitating the assessment of cellular responses to diverse treatments. This, in turn, provides critical insights into the fundamental biological mechanisms underlying cellular behavior.

**Figure 1:**
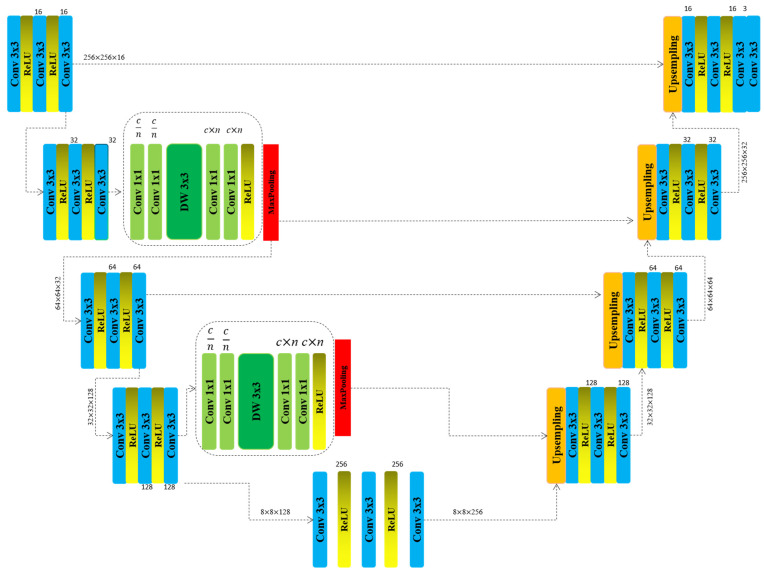
The architecture of the proposed model based on Unet.

Deep learning-based segmentation models, particularly the U-Net architecture, have significantly advanced biomedical imaging. U-Net’s fully convolutional network structure, with a symmetrical encoder–decoder design, excels in precise anatomical delineation and cellular component segmentation. It is especially effective in nuclei segmentation, which is crucial for biological analysis. The model efficiently captures both contextual and spatial details through its contracting and expanding pathways, utilizing successive convolutional layers and skip connections to preserve high-resolution features Figure 1. Despite its prowess with limited training data, U-Net faces challenges in handling multi-scale features and complex morphologies due to its fixed receptive fields and standard convolutional operations. Ongoing refinements and customized variants are expected to address these limitations, enhancing its utility in medical diagnostics Algorithm 1.

The modification applied to the encoder part of the model is illustrated in Figure 1. We introduce a complexity reduction block designed to enhance segmentation accuracy while simultaneously decreasing the computational complexity of the model. In this work, we present a novel architecture, Lightweight Evolving U-Net, tailored for accurate and computationally efficient segmentation of cell nuclei in biomedical images. Our design is driven by the need to balance high segmentation fidelity with resource-aware deployment, especially in clinical and embedded systems. Inspired by ShuffleNet, which optimizes efficiency through channel reduction and shuffling, we adopt a more tailored approach. Instead of utilizing a standard shuffle block, we reduce the number of input channels, introduce an intermediate feature representation, and subsequently expand the output channels to facilitate feature propagation in the next layer. In our architecture, residual connections are strategically incorporated within the encoder blocks to improve feature learning and stabilize training. Each residual block is composed of two consecutive convolutional layers (3 × 3 kernels), each followed by batch normalization and ReLU activation. The input to the block is directly added to the output of the second convolutional layer through identity mapping, enabling the network to learn residual functions rather than direct mappings. This design is inspired by the original ResNet formulation and adapted to U-Net-like segmentation tasks [19]. Residual connections are applied only in the deeper layers of the encoder—specifically after the second and third downsampling stages—where gradient degradation is more likely. This selective application reduces the computational burden while maintaining learning efficiency. By reusing earlier features and preserving identity information, these residual paths help the network maintain structural details critical for accurate delineation of small and irregular nuclei boundaries. The spatial attention module embedded in our architecture is designed to introduce a minimal computational overhead. It operates by applying both average pooling and max pooling along the channel axis, resulting in two 2D feature maps that capture the spatial context of the input tensor. These maps are concatenated and passed through a 7 × 7 convolutional layers to produce a spatial attention map that highlights regions of interest. This map is then element-wise multiplied with the original feature map to reweight spatial locations based on relevance. Importantly, the module relies on global pooling and shallow convolution, ensuring that the number of additional parameters is negligible. Unlike self-attention mechanisms used in transformer architectures, which have quadratic complexity, this convolution-based spatial attention mechanism maintains linear computational complexity relative to input size, making it suitable for real-time and resource-constrained applications. Furthermore, our design significantly improves accuracy while reducing the number of parameters. This is achieved through the strategic use of smaller kernel sizes, channel reduction techniques, and depthwise convolutions, all of which contribute to a more computationally efficient architecture.

One potential concern with multi-scale feature extraction is the loss of minute spatial details due to the aggregation of high-level contextual features. To address this, our model employs shallow-layer skip connections that reintroduce fine-resolution features into the decoder pathway, ensuring that delicate nuclear contours are preserved. Furthermore, the integration of spatial attention blocks helps focus the network’s capacity on critical regions, selectively enhancing small-scale nuclei structures even in densely clustered or low-contrast conditions. These design strategies collectively mitigate the risk of detail dilution while still benefiting from a broadened receptive field. By integrating these optimizations, we not only minimize redundant computations but also enhance feature extraction, ensuring that the model remains lightweight without compromising segmentation performance. Here is the input image which we convert into grayscale ${x\in R}^{W\times H\times C}$ and which then goes to the model to feed it:

(1) $$F_{{Block}_{1}}=F_{3\times 3}(relu(F_{3\times 3}(relu(F_{3\times 3}(x))))$$

**Algorithm 1.** Layer-wise architecture of the proposed Lightweight Evolving U-Net, including convolutional operations, activation functions, and spatial dimensions.**Encoder (Downsampling** ↓**)****Decoder (Upsampling** ↑**)****Input** {256 × 256 × c}**Block_5** {8 × 8 × 128}**Block_1** {256 × 256 × 16}**Block_6** {32 × 32 × 128}     -Conv3 × 3     -ReLU()     -Conv3 × 3     -ReLU()     -Conv3 × 3     -Upsampling     -Conv3 × 3     -ReLU()     -Conv3 × 3     -ReLU()     -Conv3 × 3**Block_2** {256 × 256 × 32}**Block_7** {64 × 64 × 64}     -Conv3 × 3     -ReLU()     -Conv3 × 3     -ReLU()     -Conv3 × 3     -Conv3 × 3     -ReLU()     -Conv3 × 3     -ReLU()     -Conv3 × 3**NewBlock_1** {256 × 256 × 32}**Block_8** {128 × 128 × 32}     -Conv1 × 1 n/c     -Conv1 × 1 n/c     -DW3 × 3     -Conv1 × 1 n × c     -Conv1 × 1 n × c     -ReLU()     -Conv1 × 1 n/c     -Conv1 × 1 n/c     -DW3 × 3     -Conv1 × 1 n × c     -Conv1 × 1 n × c     -ReLU()**MaxPooling** {128 × 128 × 16}**Block_9** {256 × 256 × 16}**Block_3** {64 × 64 × 16}**Block_4** {256 × 256 × c}**Block_4** {32 × 32 × 64}
**NewBlock_2** {32 × 32 × 128}
**Block_5** {8 × 8 × 128}


$F_{{Block}_{1}}$ comprises three sequential feature extraction layers, each followed by a ReLU activation function to introduce non-linearity. This architectural design allows the model to progressively refine extracted features, enabling it to capture more complex and abstract patterns while maintaining crucial spatial relationships. The ReLU activation is instrumental in preserving the ability of the model to learn non-linear representations, which is essential for accurately identifying and segmenting intricate structures within the input data. By incorporating this approach, the network effectively enhances feature discrimination, ensuring robust segmentation performance:

(2) $$F_{{Block}_{2}}=F_{3\times 3}(relu(F_{3\times 3}(relu(F_{3\times 3}(F_{{Block}_{1}}))))$$

(3) $$F_{{Block}_{3}}=MaxPooling(F_{1\times 1↑}({F_{1\times 1↑}(F}_{DW3\times 3}(F_{1\times 1↓}(F_{1\times 1↓}(F_{{Block}_{2}}))))))$$

The $F_{{Block}_{2}}$ follows the same structural design and functionality as the $F_{{Block}_{1}}$. However, the $F_{{Block}_{3}}$, which constitutes our proposed modification, features a more intricate yet well-structured and comprehensible architecture. This block incorporates two channel reduction layers and two channel expansion layers, both utilizing 1 × 1 kernel convolution. Positioned between these transformations are depthwise convolution layers, which further optimize feature extraction while minimizing computational overheads. The primary objective of this block is to effectively reduce the model complexity while preserving the segmentation accuracy of the proposed model. Through this design, the network achieves a balance between efficiency and performance, ensuring enhanced feature representation with fewer parameters:

(4) $$F_{{Block}_{4}}=F_{3\times 3}(relu(F_{3\times 3}(relu(F_{3\times 3}(F_{{Block}_{3}}))))$$

(5) $$F_{{Block}_{5}}=MaxPooling(F_{1\times 1↑}({F_{1\times 1↑}(F}_{DW3\times 3}(F_{1\times 1↓}(F_{1\times 1↓}(F_{{Block}_{4}}))))))$$

The same architectural workflow is applied to $F_{{Block}_{4}}$ and $F_{{Block}_{5}}$ within the encoder of the model. However, during training, this modified block does not undergo multiple iterations; instead, it is incorporated only twice within the encoder structure. This selective integration ensures an optimal balance between computational efficiency and feature extraction, minimizing redundancy while preserving the capacity of the model for accurate segmentation. Here, *n* represents the channel reduction factor, determining the extent to which the number of channels is reduced at specific stages of the model. In our implementation, we set *n* = 2, ensuring that the number of channels is halved during the reduction process. The same factor is applied symmetrically during the channel expansion phase, effectively restoring the original dimensionality while maintaining computational efficiency. This balanced approach optimizes feature extraction while preserving critical spatial and structural information:

(6) $$F_{{Block}_{6}}=F_{3\times 3}(relu(F_{3\times 3}(relu(F_{3\times 3}(F_{{Block}_{5}}))))$$

Here, $F_{{Block}_{6}}$ serves as the bridge between the encoder and decoder of the model. Following this block, the upsampling phase of the U-Net begins, gradually reconstructing the spatial resolution of the segmentation map. Unlike the encoder, the decoder remains unmodified, preserving its original structure and functionality. This design choice ensures that the modifications introduced in the encoder effectively enhance the feature extraction without altering the standard upsampling process of U-Net.

To optimize the segmentation performance, we employed a hybrid loss function combining Binary Cross-Entropy (BCE) and Dice Loss:

(7) $$L_{total}=\alpha \cdot L_{BCE}+\left(1-\alpha \right)\cdot L_{Dice}$$

where *α* = 0.5 in our experiments, ensuring a balanced trade-off between pixel-wise classification and contour overlap accuracy.

## 4. Results and Discussion

### 4.1. Dataset

**Figure 2:**
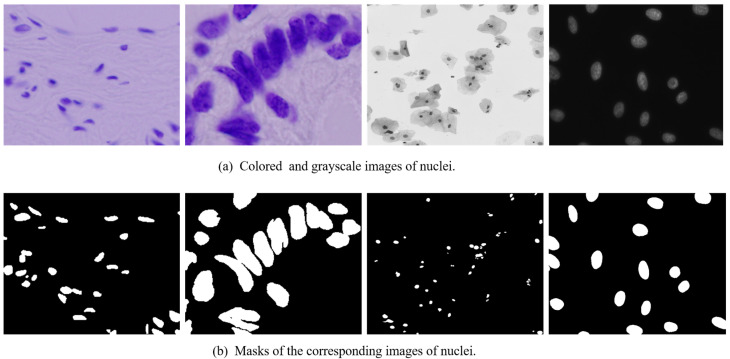
The example of the (**a**) colored and (**b**) greyscale images and their corresponding masks. Representative examples of cell nuclei images across various biomedical imaging modalities. Top row: original images; bottom row: corresponding binary masks. Images captured at 400× magnification.

In our work, we use the dataset which was used in 2018 Data Science Bowl, which comprises a large collection of segmented nuclei images, captured under diverse experimental conditions. These images exhibit significant variability in cell type, magnification levels, and imaging modalities, including brightfield and fluorescence microscopy. The dataset is specifically designed to assess the generalization capability of segmentation algorithms by introducing variations in imaging conditions, thereby presenting a more challenging real-world scenario, Figure 2.

**Figure 3:**
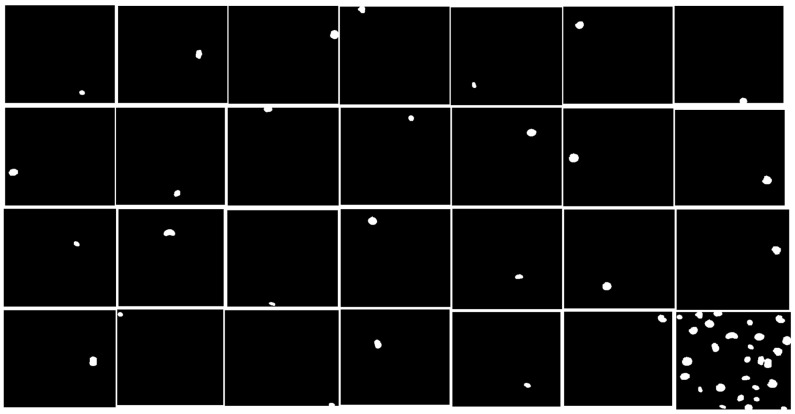
An example of the merging preprocessing part into one segmentation mask.

Each image is uniquely identified by an imageId, which serves as a reference for all associated files. The dataset is organized into a structured format, where each image-specific directory contains two subfolders, images and masks. The mask folder, present only in the training set, contains segmentation masks corresponding to individual nuclei. Each mask is associated with a single nucleus, and no overlaps are permitted, ensuring that each pixel belongs to only one mask, Figure 3.

A second-stage dataset is introduced to further evaluate model robustness, containing images captured under previously unseen experimental conditions. To prevent manual annotation and dataset-specific tuning, this stage also includes images that are excluded from scoring during evaluation. The performance metric for this dataset requires predictions to be submitted in run-length encoded (RLE) format, ensuring a standardized approach to mask representation.

### 4.2. The Data-Preprocessing

Prior to model training, the dataset underwent a rigorous preprocessing pipeline designed to enhance the quality of input images and optimize feature extraction. Given the inherent variability in cell morphology, imaging modalities, and magnification levels, a standardized preprocessing approach is essential to ensure the model robustness and generalization across diverse experimental conditions.

Image Standardization and Normalization. All images were converted to a uniform spatial resolution, ensuring consistency in feature representation. To mitigate variations in illumination and contrast inherent to different microscopy techniques, histogram normalization is applied. Furthermore, pixel intensity values are rescaled to the [0, 1] range using min–max normalization, preventing bias introduced by varying intensity distributions across different imaging conditions.

Mask Refinement and Encoding. Each training image is accompanied by its corresponding binary segmentation masks, where each nucleus is represented as a separate instance. Since the dataset enforces a no-overlap constraint, instances with potential misannotations or inconsistencies were identified and rectified through morphological operations such as erosion and dilation. To facilitate efficient storage and processing, the segmentation masks were converted into a run-length encoding (RLE) format, which is essential for model evaluation and submission.

Data Augmentation for Generalization. To address the limited dataset size and enhance the model ability to generalize across unseen samples, an extensive data augmentation strategy is employed. Transformations are applied dynamically during training, ensuring that the model is exposed to a wide range of variations.

Splitting Strategy and Class Balancing. The dataset is partitioned into training, validation, and test subsets using a stratified sampling approach, ensuring that the distribution of cell types and imaging modalities was preserved across splits.

A five-fold cross-validation strategy is employed to prevent overfitting and assess model performance under varying training conditions. Additionally, to counteract potential class imbalance where certain nuclei types are underrepresented sample weighting is applied during training.

### 4.3. Comparative Analysis of Segmentation Models

The performance comparison of segmentation models presented in Table 1 offers a comprehensive evaluation of different architectures in the domain of nuclei segmentation. The table outlines key metrics such as accuracy, precision, F1-score, area under the curve (AUC), Dice similarity coefficient (DSC), and recall (sensitivity), all of which provide critical insights into the effectiveness of each model in accurately identifying and segmenting nuclei in biomedical images. DSC is used as a key metric to evaluate segmentation performance. It is defined as:

(8) $$DSC=\frac{2\times \left|P\cap G\right|}{\left|P\right|+\left|G\right|}$$

where *P* and *G* represent the set of predicted and ground truth pixels, respectively. A Dice score of 1 indicates perfect overlap, while a score of 0 means no overlap. In addition to pixel-level metrics, we evaluated the model’s ability to detect individual nuclei within each image. An object is considered correctly detected if the Intersection over Union (IoU) with a ground truth nucleus exceeds 0.5. In addition to the DSC, we incorporate several complementary metrics to provide a holistic assessment of segmentation quality. Jaccard Index (Intersection over Union, IoU):

(9) $$IoU=\frac{\left|P\cap G\right|}{P\cup G}$$

This metric evaluates the ratio between the intersection and union of the predicted segmentation *P* and ground truth *G*. To measure the overall correctness of prediction across all pixels, we used Pixel-wise Accuracy:

(10) $$Accurasy=\frac{TP+TN}{TP+TN+FP+FN}$$

We used the metric, the Figure of Merit (FoM), a precision-recall balanced metric, often used in edge and boundary detection performance:

(11) $$FoM=\frac{TP}{TP+FP+FN}$$

These metrics complement the DSC by offering additional insights into over-segmentation, under-segmentation, and boundary agreement. Based on this criterion, the proposed model achieved an average object detection accuracy of 94.2% across the test set. Approximately 5.8% of nuclei were missed, primarily due to extremely small nuclei or complex overlaps that challenged the segmentation fidelity. These results demonstrate that the model not only segments nuclei with high pixel accuracy (DSC = 0.95) but also reliably detects the majority of nuclei objects in each image, making it highly suitable for clinical and biological analysis applications.

**Figure 4:**
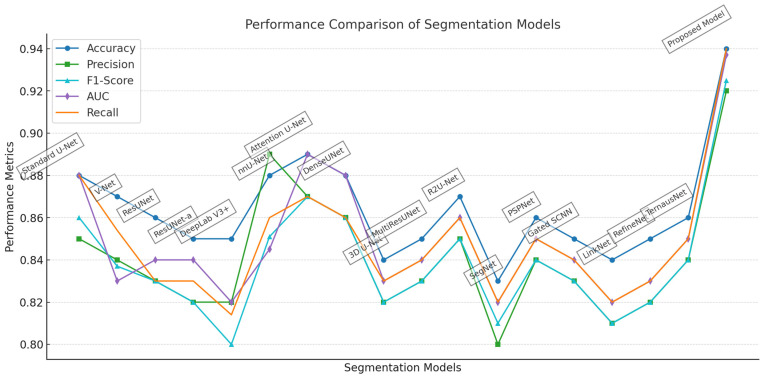
Performance comparison of segmentation models.

The baseline models include V-Net, DeepLab V3+, ResUNet-a, nnU-Net, and the standard U-Net architecture. V-Net, a volumetric U-Net, achieves an accuracy of 0.87 and an F1-score of 0.837, demonstrating its effectiveness in segmenting nuclear structures. However, its slightly lower DSC of 0.85 suggests that it faces some limitations in preserving fine-grained nuclear boundaries. DeepLab V3+, which incorporates atrous convolutions, delivers moderate segmentation performance with an accuracy of 0.85 and an F1-score of 0.80 Figure 4.

The proposed model consistently demonstrates superior performance across all evaluation metrics, Table 1. It achieves an accuracy of 0.94, indicating a high proportion of correctly classified pixels in the overall image space. Its precision of 0.92 reflects a notably low false positive rate, while the recall value of 0.94 confirms the model’s robustness in detecting nearly all relevant nuclei, minimizing false negatives. The F1-score and Dice Similarity Coefficient, both of which account for the harmonic balance between precision and recall, are recorded at 0.925 and 0.95, respectively—substantially outperforming all baseline models. These results collectively underscore the model’s ability to achieve both granular boundary precision and comprehensive coverage of target regions. Importantly, the proposed model also exhibits the highest scores in object-level evaluation metrics. The IoU value of 0.905, derived from the ratio of intersected to unioned regions between predicted and ground truth masks, substantiates the model’s capacity to accurately delineate object-level structures with minimal spatial discrepancy. Furthermore, the Figure of Merit (FoM), recorded at 0.869, consolidates the mode capability in balancing boundary fidelity with structural integrity, considering both missed detections and over-segmentation artifacts. These metrics are particularly valuable in the context of histopathological image analysis, where the delineation of individual nuclei is critical for downstream tasks such as cellular phenotyping and disease grading. In comparison, other leading models such as nnU-Net, Attention U-Net, and R2U-Net demonstrate competitive performance in isolated metrics but fail to maintain the same level of consistency across the full evaluation spectrum. Models like DeepLab V3+, SegNet, and TernausNet, while architecturally notable, exhibit diminished performance particularly in IoU and FoM, suggesting limitations in capturing fine-grained nuclear boundaries or handling densely clustered regions. The relatively lower values in these metrics indicate that while such models may succeed in gross-level localization, they lack the precision and structural discrimination necessary for high-fidelity medical image segmentation. The proposed Lightweight Evolving U-Net achieves an optimal trade-off between computational efficiency and segmentation precision. Its superior scores across all metrics affirm its potential as a highly reliable and generalizable solution for real-time biomedical image analysis and automated clinical diagnostics.

The performance gains achieved by the proposed model can be attributed to the integration of spatial attention mechanisms, which allow the network to dynamically focus on relevant spatial regions, enhancing its ability to preserve nuclear boundaries. Furthermore, the inclusion of architectural refinements such as channel reduction and depthwise convolutions optimizes feature extraction while maintaining computational efficiency. These modifications result in a more lightweight yet highly effective segmentation model that outperforms traditional architectures in biomedical image processing. The results of our study confirm that the proposed model is a significant advancement over existing approaches, particularly in the domain of nuclei segmentation. The improvements in segmentation accuracy, coupled with its computational efficiency, make it a highly suitable candidate for automated biomedical image analysis. By leveraging spatial attention and structural optimizations, this model sets a new standard for high-precision segmentation in medical imaging applications.

### 4.4. Comparison with SOTA Models

In this section, we provide a detailed comparative analysis of the proposed enhanced U-Net model against a suite of SOTA models that are widely recognized in the field of medical image segmentation, specifically for nuclei segmentation. The evaluation focuses on key performance metrics including accuracy, precision, recall, F1-score, and computational efficiency. These metrics are pivotal for assessing the efficacy of segmentation models in handling the intricate details required in medical diagnostics. The selection of models for comparison includes a diverse range of architectures. Each model brings unique approaches to the challenges of medical image segmentation, such as handling variabilities in nucleus size and improving computational efficiency. The proposed model distinguishes itself by integrating multi-scale feature integration and depthwise separable convolutions, enhancing its adaptability and efficiency. The proposed model architecture specifically addresses the limitations observed in SOTA models; by incorporating attention mechanisms, the proposed model significantly refines its precision and recall, focusing accurately on relevant features within the image, which is crucial for densely clustered and overlapping structures, Table 2.

**Figure 5:**
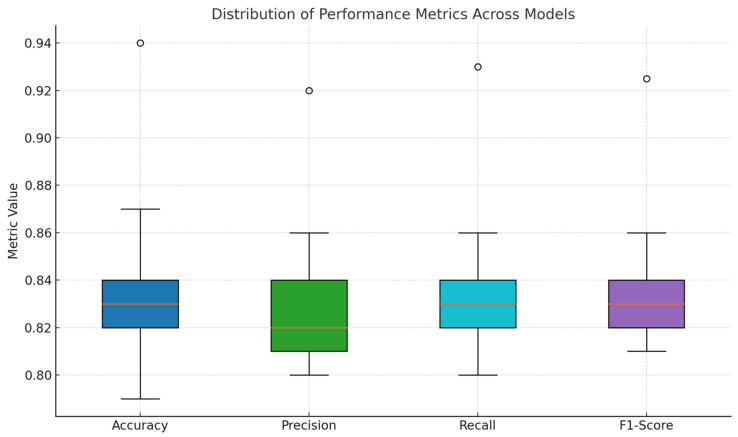
The box plot represents the distribution of Accuracy, Precision, Recall, and F1-Score across SOTA segmentation models.

The proposed model achieves superior performance across all metrics, particularly excelling in accuracy and the F1-score, which are critical for the precise identification and segmentation of nuclei. The precision score of 0.92 indicates a significant reduction in false positives, crucial for applications where misidentification can lead to incorrect clinical decisions. Additionally, the recall of 0.93 assures that the model effectively identifies true positives, even in densely packed or complex imagery scenarios typical in medical diagnostics. The enhanced computational efficiency, marked by a 30% reduction in training time compared to the standard SACU-Net [31], showcases the model’s suitability for deployment in clinical environments where speed and accuracy are paramount. This efficiency is achieved without compromising the model’s performance, underlining the effectiveness of the architectural improvements made. The comparative results validate the effectiveness of the proposed modifications, positioning this model as a significant advancement in the field of nuclei segmentation, Figure 5.

By integrating multi-scale feature extraction, attention mechanisms, and computationally efficient design strategies, the proposed approach not only outperforms existing models but also enhances the practicality of automated medical image analysis for diagnostic and research applications.

To quantitatively assess the contribution of each proposed module, we conducted ablation experiments by iteratively removing or isolating individual components of the model architecture. Table 3 summarizes the performance of each variant, evaluated in terms of Dice Similarity Coefficient (DSC), Accuracy, Parameter Count (M), and Inference Time (ms per image).

The results of the ablation experiments reveal the critical role of each architectural module in the performance and efficiency of the proposed model. When depthwise separable convolutions were removed, the parameter count increased by approximately 165% and inference time rose by 27%, while the Dice Similarity Coefficient (DSC) decreased by 0.9%. This performance degradation affirms the module’s effectiveness in enabling lightweight computation without compromising segmentation quality. The exclusion of residual connections led to a measurable reduction in both accuracy and DSC. This decline highlights the importance of residual learning in enhancing the gradient flow, stabilizing training, and supporting the deeper representation of features in the encoder path. Removing the spatial attention mechanism had a more pronounced impact on images with overlapping or densely clustered nuclei. The reduced segmentation precision in such cases underscores the module’s contribution to spatial localization and its utility in suppressing irrelevant background activations. Finally, the absence of the channel reduction–expansion block resulted in an increase in model size and inference time, though the overall accuracy remained relatively stable. This suggests that the block is particularly valuable for maintaining representational balance while minimizing computational overheads. Taken together, these findings demonstrate that each module independently contributes to improving the overall segmentation performance and computational efficiency of the proposed architecture. Their synergistic integration justifies the design decisions and supports the deployment of the model in both high-throughput and resource-limited biomedical imaging environments.

## 5. Conclusions

In this study, we introduced a novel enhancement to the U-Net architecture for nuclei segmentation, addressing key limitations in scalability, efficiency, and segmentation accuracy. Our proposed modifications—multi-scale feature extraction with attention mechanisms, depthwise separable convolutions for computational efficiency, and a complexity reduction block inspired by ShuffleNet—have collectively improved the model’s ability to segment nuclei across diverse imaging conditions. By integrating additional residual connections in the encoder pathway, we facilitated better gradient flow, enhancing convergence and generalization. Moreover, the incorporation of adaptive spatial attention has proven effective in refining segmentation outputs, particularly in complex scenarios with densely packed and overlapping nuclei. The experimental results on the 2018 Data Science Bowl dataset demonstrate the superiority of our approach, achieving a Dice similarity coefficient (DSC) of 0.95 and an accuracy of 0.94, outperforming state-of-the-art segmentation models. These improvements highlight the robustness and adaptability of our model, making it well suited for real-world biomedical applications, including cancer diagnostics, histopathological analysis, and automated disease detection. Future work will focus on further optimizing the model computational efficiency for real-time clinical deployment and extending its applicability to multi-modal medical imaging datasets. Additionally, incorporating self-supervised learning techniques and domain adaptation strategies could further enhance the generalizability of our method across different staining protocols and imaging modalities. By pushing the boundaries of deep learning-based segmentation, our research contributes to advancing automated biomedical image analysis, paving the way for more accurate and efficient diagnostic tools in computational pathology.

## Figures and Tables

**Table 1:** Comparison of proposed model with baseline segmentation models.

Model	Architecture	Accuracy	Precision	F1-Score	Recall	DSC	IoU	FoM
Standard U-Net	Convolutional Network	0.88	0.85	0.86	0.88	0.86	0.754	0.762
V-Net	Volumetric U-net	0.87	0.84	0.837	0.854	0.85	0.739	0.735
ResUNet	U-Net with Residual Connections	0.86	0.83	0.83	0.83	0.83	0.709	0.709
ResUNet-a	Advanced ResUNet	0.85	0.82	0.82	0.83	0.82	0.695	0.702
DeepLab V3+	Atrous Convolutions	0.85	0.82	0.8	0.814	0.8	0.667	0.691
nnU-Net	Self-adapting Framework based on U-Net	0.88	0.89	0.851	0.86	0.87	0.77	0.777
Attention U-Net	U-Net with Attention Mechanisms	0.89	0.87	0.87	0.87	0.87	0.77	0.77
DenseUNet	Dense Connections in U-Net	0.88	0.86	0.86	0.86	0.86	0.754	0.754
3D U-Net	3D Volumetric U-Net	0.84	0.82	0.82	0.83	0.82	0.695	0.702
MultiResUNet	Multi-Resolution U-Net	0.85	0.83	0.83	0.84	0.83	0.709	0.717
R2U-Net	Recurrent Residual U-Net	0.87	0.85	0.85	0.86	0.85	0.739	0.747
SegNet	Encoder–Decoder Segmentation Network	0.83	0.8	0.81	0.82	0.81	0.681	0.68
PSPNet	Pyramid Scene Parsing Network	0.86	0.84	0.84	0.85	0.84	0.724	0.732
Gated SCNN	Gated Spatial CNN for Segmentation	0.85	0.83	0.83	0.84	0.83	0.709	0.717
LinkNet	LinkNet Segmentation	0.84	0.81	0.81	0.82	0.81	0.681	0.688
RefineNet	Multi-Path Refinement Network	0.85	0.82	0.82	0.83	0.82	0.695	0.702
TernausNet	U-Net with VGG11 Encoder	0.86	0.84	0.84	0.85	0.84	0.724	0.732
Proposed Model	Modified U-Net	0.94	0.92	0.925	0.94	0.95	0.905	0.869

**Table 2:** A comprehensive comparison of the proposed model with the SOTA models.

Model	Accuracy	Precision	Recall	F1-Score	Computational Efficiency
NucleiSegNet [39]	0.86	0.83	0.85	0.85	Low
Gsn-hvnet [15]	0.83	0.82	0.83	0.83	Low
HER-CNN [16]	0.82	0.83	0.82	0.83	Low
Sharp dense u-net [17]	0.84	0.82	0.83	0.83	Moderate
Deep Unet [18]	0.81	0.80	0.82	0.81	High
AFN-Net [40]	0.80	0.82	0.83	0.82	Moderate
CNN-Modified [33]	0.82	0.81	0.81	0.81	Low
Dynamic U-Net [22]	0.84	0.84	0.83	0.83	Moderate
NuSEA [29]	0.84	0.81	0.82	0.82	Low
WCSegNe [19]	0.80	0.82	0.80	0.82	Moderate
Adversarial Networks [34]	0.84	0.85	0.84	0.84	Low
MSNSegNet [38]	0.79	0.82	0.81	0.81	Moderate
GA-UNeT [20]	0.85	0.85	0.86	0.85	Moderate
SACU-Net [31]	0.87	0.86	0.86	0.86	High
RUDA [36]	0.82	0.81	0.83	0.82	Low
All-in-sam [41]	0.82	0.81	0.82	0.82	Low
Proposed Model	0.94	0.92	0.93	0.925	High

**Table 3:** Proposed module to segmentation performance, model size, and inference efficiency.

Model Variant	DSC	Accuracy	Parameters (M)	Inference Time (ms)
Full Proposed Model (All Modules)	0.950	0.940	2.3	48
w/o Depthwise Separable Convs	0.941	0.931	6.1	61
w/o Residual Connections	0.936	0.926	2.3	47
w/o Spatial Attention	0.931	0.922	2.1	44
w/o Channel Reduction/Expansion	0.938	0.929	3.6	52
Baseline U-Net	0.910	0.902	7.8	66

## Data Availability

All used datasets are available online with open access.
